# Linking Pathological Oscillations With Altered Temporal Processing in Parkinsons Disease: Neurophysiological Mechanisms and Implications for Neuromodulation

**DOI:** 10.3389/fneur.2019.00462

**Published:** 2019-05-10

**Authors:** Martijn Beudel, Anna Sadnicka, Mark Edwards, Bauke M. de Jong

**Affiliations:** ^1^Department of Neurology, Amsterdam Neuroscience Institute, Amsterdam University Medical Center, Amsterdam, Netherlands; ^2^Department of Neurology, University Medical Center Groningen, University of Groningen, Groningen, Netherlands; ^3^Faculty of Brain Sciences, Institute of Neurology, University College London, London, United Kingdom; ^4^Department of Neurology, St. George's University of London, London, United Kingdom

**Keywords:** Parkinson's disease, deep brain stimulation, oscillations, timing, movement disorders

## Abstract

Emerging evidence suggests that Parkinson's disease (PD) results from disrupted oscillatory activity in cortico-basal ganglia-thalamo-cortical (CBGTC) and cerebellar networks which can be partially corrected by applying deep brain stimulation (DBS). The inherent dynamic nature of such oscillatory activity might implicate that is represents temporal aspects of motor control. While the timing of muscle activities in CBGTC networks constitute the temporal dimensions of distinct motor acts, these very networks are also involved in somatosensory processing. In this respect, a temporal aspect of somatosensory processing in motor control concerns matching predicted (feedforward) and actual (feedback) sensory consequences of movement which implies a distinct contribution to demarcating the temporal order of events. Emerging evidence shows that such somatosensory processing is altered in movement disorders. This raises the question how disrupted oscillatory activity is related to impaired temporal processing and how/whether DBS can functionally restore this. In this perspective article, the neural underpinnings of temporal processing will be reviewed and translated to the specific alternated oscillatory neural activity specifically found in Parkinson's disease. These findings will be integrated in a neurophysiological framework linking somatosensory and motor processing. Finally, future implications for neuromodulation will be discussed with potential implications for strategy across a range of movement disorders.

## Time and the Brain

Purposeful motor behavior requires adequate spatio-temporal co-ordination of limbs and axial body parts. E.g., in catching a ball, joint movements, are arranged in such a way that the hand reaches the target's location at the correct time. These spatial and temporal characteristics of movement are also distinctively represented in the organization underlying cerebral motor control, embedded in both segregated and integrated neuronal circuitries (1, 2). Regarding motor timing, at least two interrelated levels of organization may be discerned:

(i) planning “to be” at the right time at the right place and

(ii) planning the serial order of required muscle contractions in order “to move” effectively. The first highlights engagement with a dynamic environment and underscores the relation between motor and perceptual timing. Given the relative slow cerebral processing time, such environmental engagement requires anticipation on near-future events. With regard to the temporal orchestration of muscle contractions, proprioception makes an additional sensory contribution to motor control, both in feedforward and feedback modes (3).

**Figure 1:**
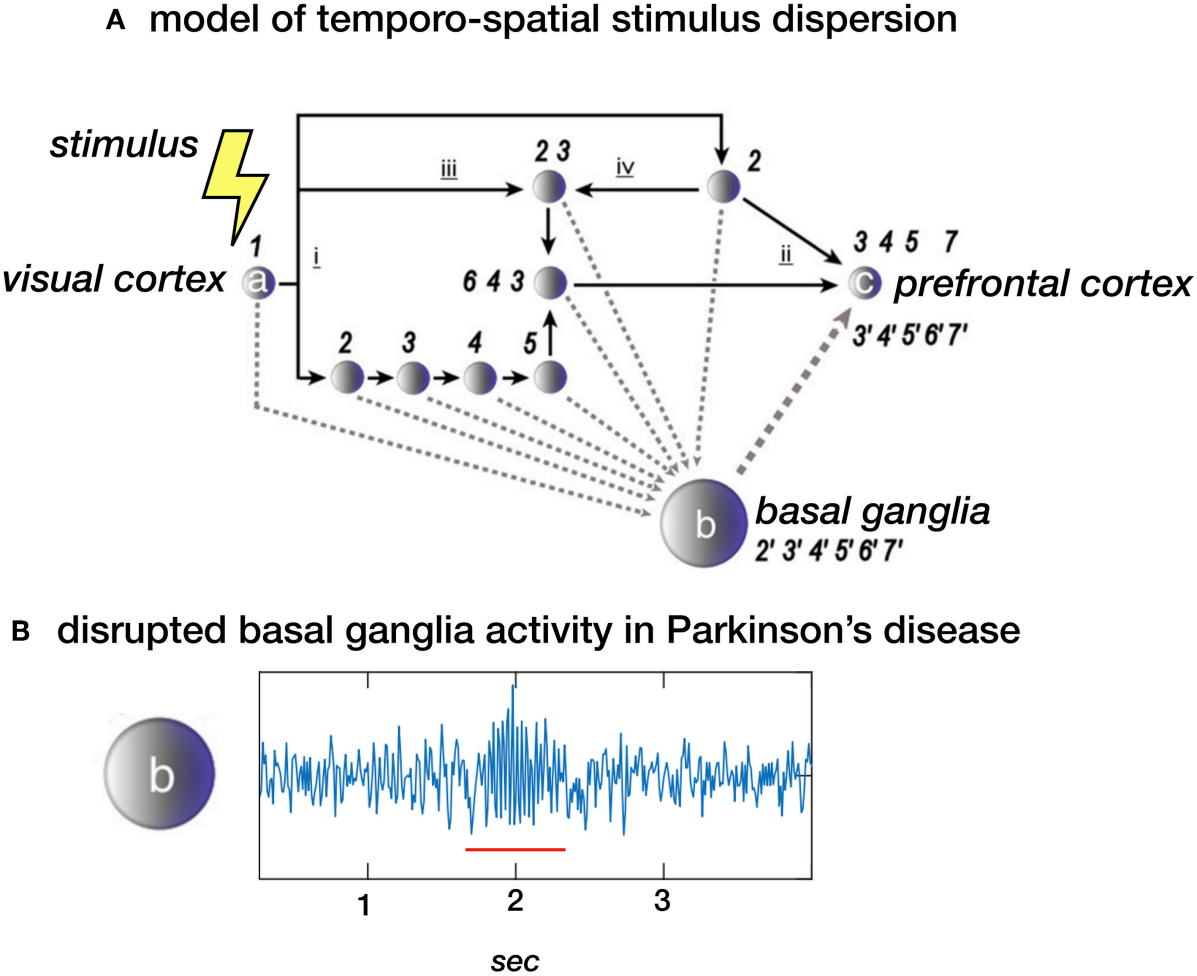
**(A)** Stimulus dispersion in space and time. Scheme of a simplified neuronal network to illustrate dispersion in space and time following the initial stimulus-induced activation (1) of e.g., the visual cortex (locus a). Successive processing steps (indicated by the numbers 2–7) take place according to principles of functional segregation (i) and integration (ii), as well as bottom-up (iii) and top-down (iv) mechanisms. Differences in synaptic delay along parallel processing streams, due to different number of processing nodes along each pathway, may introduce sequence irregularity. At system-level, however, sequential regularity is maintained by the integration of efferent copies sent to the basal ganglia (locus b). The latter may act as an oscillator, providing a measure of “processing-based” time at network locus c (e.g., the prefrontal cortex). **(B)** Local field potential (LFP) showing neural activity in the subthalamic nucleus (STN) in a healthy subject of a patient with Parkinsons disease. The “burst” with increased and more synchronized activity in the beta (13–30 Hz) domain (underscored with a red line) is typical for Parkinsons disease. In theory, the proposed model depicted in *a*. could be disturbed by this pathological activity. **(A)** Derived from (5), NeuroImage **(B)** unpublished data.

The dynamics of an environment, i.e., the succession of events, is perceived as a sequence of spatial changes. Segregated analysis of the various features of (visual) stimuli, such as shape, color, and visual motion, along parallel processing streams with varying synaptic delays, implies that the initial stimulus time is dispersed within the brain, thus losing an absolute (external) measure of time (4, 5). Moreover, a consequence of such necessary cerebral processing time is that the flow of external change is fractionated, demarcating intervals between distinct spatial frames. This is consistent with daily-life experience that at certain speed, a moving dot will appear as a line. Apparently, intervals of minimal change define the threshold at which a moving dot is either perceived as a dot or changes into a line. Based on functional imaging results the cerebellum is particularly implicated in assessing differences between past and future spatial frames enabling anticipation on coming events (6, 7), while the striatum plays a specific role in monitoring minimal intervals of successive spatial change, providing an internal measure of non-contextual time, i.e., an internal clock (5, 8, 9). For the latter, parallel streams of cortical processing steps, with the intrinsic consequence of introducing temporal dispersion, efferent copies might be emitted to the striatum and provide sequential regularity at system level (Figure 1). This is consistent with the model of cortical networks that enable “timing” as a result of time-dependent network changes (10). Closely related to this concept, the basal ganglia have been proposed to play a role in the synchronization of multiple cortical oscillators (11, 12).

The concept of minimal intervals of spatial change finds particularly support from a variant of the “flash-lag illusion,” i.e., a “flash-lead” illusion. This phenomenon implies that a moving object is perceived to be behind a spatially concurrent stationary flash before the two disappear (13). Disappearance of the moving dot interrupts building the final “frame,” leaving perception of the preceding frame registering its foregoing location. Assuming that the delay reflects processing time required to construct a “single spatial frame,” it was speculated that this temporal measure is in the magnitude of 100 ms. In the classical “flash-lag” illusion, which implied that the moving object did not disappear but proceeded its trajectory, the moving object is perceived to be ahead of the spatially concurrent stationary flash (14, 15). Here, it is proposed that the percept (“spatial frame”) attributed to the time of the flash is a function of events that happen in the ~100 ms after the flash, i.e., the processing time to construct such frame, with interpolation of the past concerning the moving object (16, 17). In Parkinson's disease (PD), this illusion is disrupted (18). In the present paper, we will discuss the specific alterations of temporal processing in PD and its implications for applying neuromodulation, in particular deep brain stimulation (DBS).

## Temporal Processing in Parkinson's Disease

One of the most remarkable features of disturbed temporal processing in PD is noticed when assessing the defining feature of PD; bradykinesia. When asked to make regular finger, hand and arm movements, PD patients show specific difficulty in maintaining sufficient velocity and regularity/rhythm. Furthermore, there is a vast amount of experimental evidence that shows that temporal processing in the perceptual domain is abnormal in PD (19–21). This not only holds for the temporal discrimination threshold (TDT)–the minimum inter-stimulus time between two sensory stimuli which a subject can reliably detect that there are two stimuli rather than one (22) but also for the perception of inter-stimulus intervals (23) and rhythm processing (24). As a consequence, perceptual illusions, such as the rubber hand (25) and flash-lag illusion (18), are perceived differently in PD patients compared to controls. After the application of dopaminergic drugs, several studies have shown a reduction of the TDT more toward healthy controls (22), similar observations are noticed after the application of DBS whereafter the discrimination of auditory inter-stimulus intervals improves (26). As temporal processing improves, recent evidence shows that also perceptual illusions like the rubber hand illusion improve after the application of DBS (27). In several studies the degree of the disturbance in temporal processing is correlated with the severity of Parkinsonian motor symptoms (18, 22, 28). These correlations link the disturbed velocity of movements with temporal processing capacities; the slower a patient moves the slower he / or she can perceive temporal changes.

The pathophysiological mechanism of the disrupted temporal processing in PD are not yet fully elucidated. The fact that there is a dopaminergic depletion in PD and that dopaminergic drugs (and DBS) restore alterations in temporal-processing made initial hypothesis about the its origin go to a “dopaminergic clock” (29). This is further supported by animal-experiments showing that drugs with an opposite effect, neuroleptics, show a decrease of the velocity of the dopaminergic clock and that this is dependent on de D2 affinity of the neuroleptic (30). Further evidence for such dopaminergic clock comes from PET imaging in which TDT values were correlated with the severity of the striatal dopaminergic deficit (31) and fMRI studies in healthy volunteers in which striatal activation occurred during temporal processing (5, 8). Although this correlation provides further evidence for a dopaminergic role. It does not yet elucidate the mechanism at a more explicit neural network level. For this reason, data with a higher temporal resolution; neurophysiological signals, can provide such evidence.

## Neurophysiological Alterations in Parkinson's Disease

Stereotactic and functional neurosurgery for movement disorders have provided a unique opportunity to directly record neural activity in the basal ganglia and cortex from either the single neuron, using micro-electrode recordings (MER), or from populations of neurons, using local field potentials (LFP). Furthermore, with advanced signal processing techniques it is currently also possible to extract EEG characteristics, that are specific for movement disorders.

In PD, an excessive domination of beta (13–30 Hz) oscillations is found throughout the motor system. Up to now, the origin of these oscillation in patients is not yet established but recent animal studies have shown that dopaminergic depletion leads to increased firing of striatal spinal projection neurons of the indirect pathway which in turn become prone to being recruited for exaggerated beta oscillations (32) These beta-oscillations decrease after the application of dopaminergic medication (33) and DBS (34). One of the limitations of these findings is that no reference values are present from healthy controls, which makes disease-specificity difficult to prove. However, the amount of beta oscillations correlates with contralateral akinesia-rigidity scores (35) and are more pronounced in PD as in dystonia in a recent meta-analysis (36). Furthermore, not only, the power of local beta oscillations but also the coherence in the beta range between cortex and STN is altered (37) and can be restored by applying DBS (38). One recent, finding is that the presence of increased beta activity changes over time and transient increases of beta activation occurs in so called “bursts” (39, 40). The longer the average burst duration is, the more severe PD symptoms are. Further evidence for a relation between symptom severity with the stability of beta oscillations over time comes from a study in which the *variability* of beta-power inversely correlated with symptom severity in PD (41). This is consistent with the implication that longer beta bursts reflect reduced beta power variability. In other words, excessive enhanced synchronization of activity in the beta band is present in PD and is correlated with the severity of motor symptoms.

## Linking Neurophysiological Alterations with Temporal Processing

**Figure 2:**
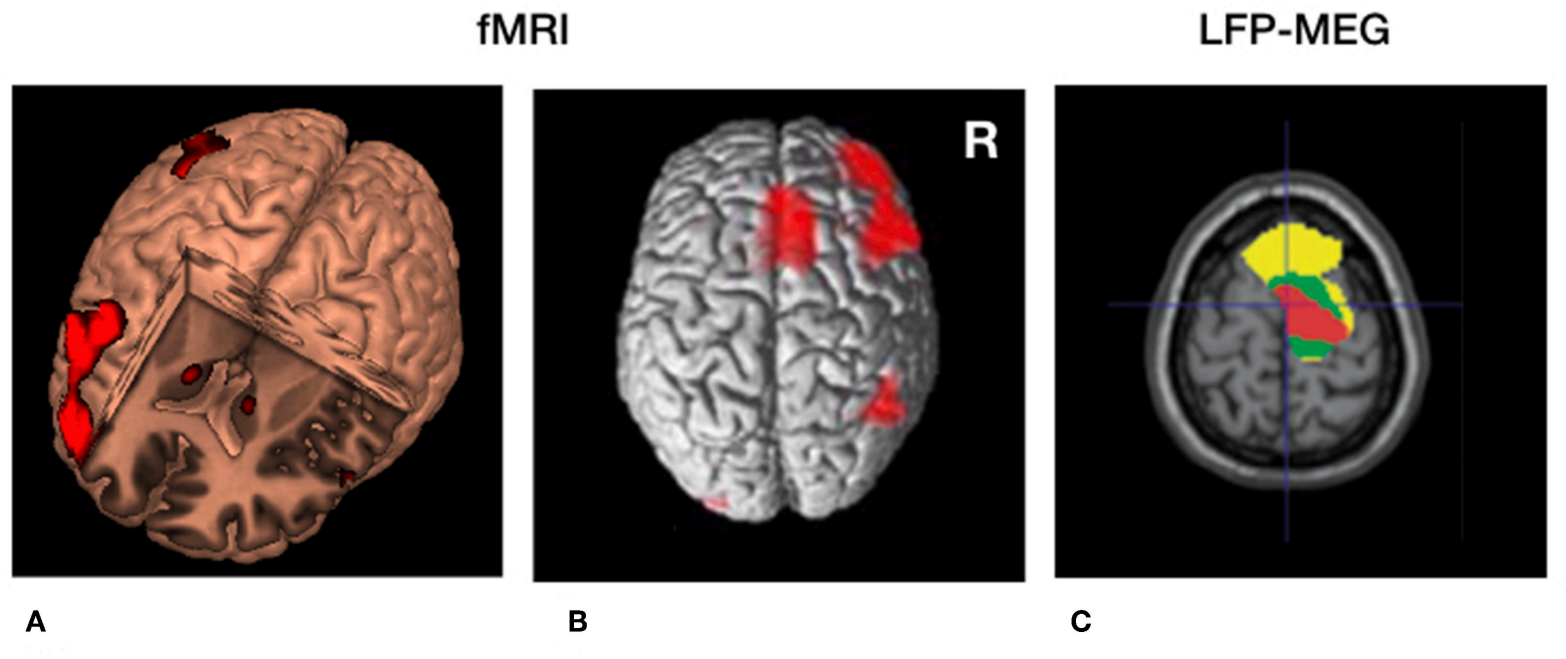
Cerebral circuitry involved in temporal processing and cerebral circuitry selectively modulated by deep brain stimulation (DBS) in Parkinson's disease. **(A)** 3D fMRI activation pattern in healthy volunteers during a temporal estimation task showing increased bilateral basal ganglia activity and right frontal activation. **(B)** Similar rendered contrast showing additional activation of the supplementary motor area (SMA). **(C)** Beta (13–30 Hz) coherence between local field potentials (LFP's) of the subthalamic nucleus (STN) and frontal regions recorded with magnetoencephalography (MEG). Yellow, low beta (13–21 Hz); Green, high beta (21–30 Hz) coherence. Red = decreased cortical beta coherence after the application of DBS. **(A,B)** Derived with permission from (5), NeuroImage **(C)** derived with permission from (38), Brain.

So far, no experimental evidence is available that directly links disturbed (beta) oscillatory activity to disturbed temporal processing in PD. However, beta oscillations have been involved in many other processes beyond movement. Transient beta oscillations play an important role in the successful stopping of actions (42, 43). Furthermore, beta oscillations play a role in “status quo maintenance” (44) and “top-down” based attention (45). Next to this, the *volatility* of beta oscillations (beta modulation) is involved in the sequencing of complex sensorimotor processes including repetitive movements (46) as well as passive listening to isochronous sounds (47). Based on these findings and the arguments presented in the previous sections one may infer that the correlation of PD symptom severity with (i) the amount of beta oscillations as well as (ii) altered perceptual time processing, provides an indirect arguments for the context that exaggerated oscillatory activity in the motor system [e.g., (38)] represents a neuronal mechanisms causing altered temporal processing in general. In other words, one may ask whether it is possible that exaggerated synchronized activity discards temporally sensitive information (efferent copies) as a noise filter (Figure 2) And consequently, is the magnitude of established change in perceptual illusions determined based on exaggerated beta oscillations? These hypotheses can be tested by combining psychophysical paradigms with time-locked neurophysiological recordings [e.g., (48)].

## Implications for Neuromodulation

**Figure 3:**
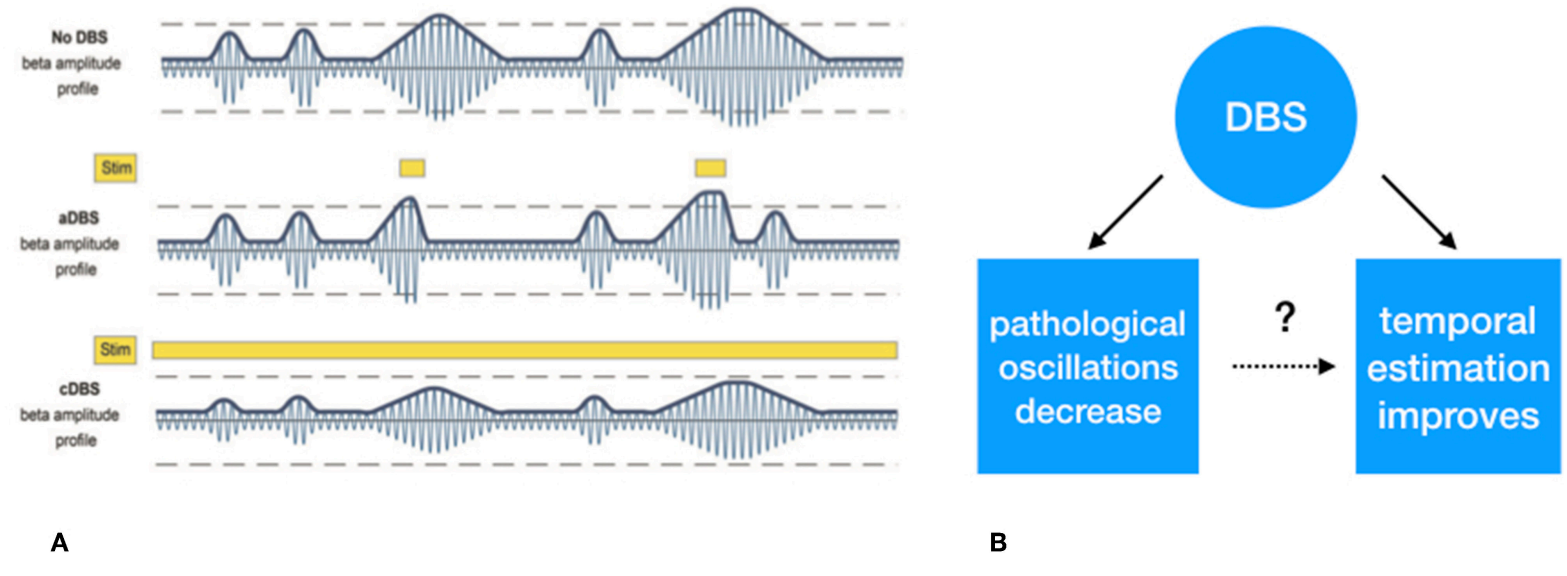
**(A)** Schematic representation of exaggerated beta (13–30 Hz) “burst” activity derived from the subthalamic nucleus. The first row depicts the similar periodically enhanced beta activity as depicted in Figure 1. The second row depicts the effect of selective suppression of beta burst activity of a certain magnitude and duration. This leads to selective, i.e., adaptive, stimulation. In the third row, the non-selective effect of continuous stimulation, conventional DBS, is depicted which leads to an overall suppression of beta activity, irrespective its magnitude, and duration. **(B)** Whether the suppression of exaggerated leads to improved temporal estimation is to be tested. **(A)** Derived with permission from (39), Brain.

Although DBS is an established treatment for refractory movement disorders, it has its limitations in terms of efficacy and side-effects. One of the reasons for this is that not only pathological but also physiological neural activity is suppressed. Given the natural fluctuation of the severity of Parkinsonian symptoms, e.g., due to dopaminergic medication and fatigue, DBS might, in PD, only be effective when pathological neural activity is present, and symptoms are present [e.g., (49)]. This implies that DBS should indeed not be provided continuously but in an intermittent and adaptive way, adaptive DBS (aDBS) (50). Up to now, all the experimental evidence for aDBS comes from paradigms in which high frequency stimulation (±130 Hz) is either switched ON or OFF or modulated in amplitude based on the amount of pathological beta oscillations (Figure 3). On the other hand, beta oscillations also fulfill a physiological, anti-kinetic role in terminating actions (43). When applying DBS in such a way that it is only switched on when disruptive beta oscillations are present, DBS might provide a more profound effect while stimulation induced side effects would be reduced (51).

Although the approach described before adapts stimulation on the amount of pathological oscillations, it is still independent of sensorimotor processing. Another approach to time neuromodulation is to apply stimulation based on the presence of *events* when pathological activation occurs. Such approach has been clinically applied for almost a decade in epilepsy patients [e.g., (52)] and recently also a patient with Tourette syndrome (53). In these two disorders DBS was triggered by the presence of epileptic activity and presence of neural activity associated with tics, respectively. In theory, DBS could also be triggered by other potentials that are, for example, related to movement initiation. This approach has recently been trialed a in a stroke recovery model (54). In this study, electrical stimulation time-locked to transient LFO's, which occur during skilled upper limb tasks, significantly improved upper limb function. Such form of precision medicine highlights the importance of the accurate timing of neuromodulation. Further support for such a phase-specific role of neuromodulation comes from tremor studies in which DBS (55) or transcranial direct current stimulation [TDCS, (49)] aligned to tremor phase, resulted in significant tremor suppression with minimally delivered energy. Although, such an “event-based” form of stimulation has not been trialed in PD, a recent paper (56) showed the temporal course of STN activation during inhibitory, motor, and cognitive tasks, which might form the basis for such stimulation algorithms. By applying these event based analyses of LFP's the effects of different forms of stimulation can be tested and a the role between physiological oscillatory activity [e.g., (48)] and disturbed oscillatory activity could be better elucidated (Figure 3). In line with this, by filtering disturbed oscillatory activity, but leaving physiological neuronal activity unaffected, sensorimotor processing, including temporal processing might be return to physiological conditions. Evidence for this comes from recent studies showing that conventional (continuous) DBS shows both a more constant suppression of subcortical (39) and cortical beta activity (57), which might lack the essential volatility to show peri-stimulus beta modulation to process essential temporal information. Of course this needs to be proven by empirical findings and we hope to test these hypotheses in the nearby future.

## Parallels within other Movement Disorders

Dystonia is another movement disorder in which temporal processing has been extensively tested. Temporal discrimination thresholds have been found to be elevated across both the visual and somatosensory domains (58). Interestingly in cervical dystonia, abnormalities in temporal discrimination in relatives has led to the hypothesis that abnormalities in TDT are a marker of non-manifesting gene carriage [acting as an endophenotype, (59)]. In contrast to PD, TDT abnormalities persist despite the efficacy of GPi-DBS, suggesting that DBS does not appear to improve dystonic motor activity by correcting abnormalities in sensory processing, at least as measured by the TDT (60). A distributed network is likely to be involved in the processing of TDT, however, notably much of the variability of TDT values across subjects has been linked to neural markers of inhibition in the primary somatosensory cortex [S1, (61)]. Furthermore, high frequency repetitive stimulation over the S1 increases neurophysiological makers of inhibition and sensory function and such changes correlate with improvements in TDT (62). Thus, similar to PD, such findings hint that neural correlates of TDT abnormalities could be used to provide a richer input environment to inform adaptive DBS strategies. This is particularly relevant in dystonia as clinical response often lags many months behind changes or the initiation of DBS offering the operator little real time feedback by which to optimize stimulation parameters.

Compared to cerebellar ataxia, as seen in cerebellar degeneration, PD patients show a selective disturbance in rhythmic temporal prediction, and not in single interval prediction (19, 21). These findings are in line with the role of the basal ganglia in monitoring minimal intervals of successive spatial change, providing an internal measure of non-contextual time (See section Time and the Brain).

## Future work

In order to further elucidate the true nature of disturbed temporal processing and its potential therapeutic consequences in PD, several new avenues are currently being explored. Testing temporal processing draws on psychophysics, and there are some drawbacks of the different psychophysical paradigms which have been previously applied. Since most of these paradigms rely on self-report, they can be subject to bias. Standard staircase methodology in which the separation between two stimuli is slowly increased or decreased in a predictable manner allows the obtained thresholds to be readily biased by a decision strategy unrelated to temporal discrimination ability (63). For example, if stimuli are gradually changed in the direction of threshold over several trials experimentally it has been shown that some observers develop a habit of repeating the same response and thus continuing to make the response after the threshold point has been reached. This “error of habituation” affects the data by falsely increasing thresholds (64). Within some paradigms catch trials (single stimuli trials) are also introduced which attempts to mitigate these errors and encourages participants to evaluate the sensory information arriving on each trial. However, such biases are best mitigated by randomizing the order of presentation (64).

The analysis of psychophysical paradigms has also progressed hugely since the methods of limits were established and their limitations acknowledged in the 1960s. By using reaction time data as well as well as accuracy of response inferences can also be made about the decision making components integral to the TDT. Such factors have begun to be explored in PD. For example, computational modeling has revealed that timing deficits in PD cannot be solely attributed to perceptual temporal distortions, but are also associated with impulsive decision strategies that bias patients toward premature responses (65). Similarly, drift diffusion decision modeling in a large group of subjects with cervical dystonia points to a more conservative decision strategy in cervical dystonia over and above a temporal discrimination deficit (63). Such finding are highly feasible as the subtle neuropsychiatric profile of many movement disorders are increasingly appreciated.

Although such studies increase the complexity of interpretation of simple TDT threshold embracing such techniques and analysis may prove its utility in the future. We are still far from having disease specific psychophysical markers for temporal processing. By better estimation of the true psychophysical deficit psychophysical deficits unique to particular disease states may be found. This yields greater power to researchers to discover the corresponding neural correlate which could be used for aDBS. This especially involves the experiments in which state of art neuromodulation, neurophysiology and psychophysics are simultaneously applied.

## Conclusion

Temporal processing is disturbed in PD while the severity of the movement disorder is sometimes correlated with the magnitude of changes in perceptual temporal processing. It is not yet established whether there is a causative link but pathological neural oscillatory activity might play a role. Furthermore, DBS improves motor performance and perceptual temporal processing and reduces pathological neural oscillatory activity. These observations provide indirect evidence that temporal processing is similarly affected by the same pathological neural oscillatory activity. As we move toward an era of more effective adaptive DBS finding neural correlates of temporal processing abnormalities may allow DBS to be dynamically titrated in response to a wider range of pathophysiological parameters. By bringing together neuromodulation, advanced neurophysiology and psychophysics, these hypotheses can be tested.

## Author Contributions

BdJ and AS respectively wrote the 1st and 6th + 7th sections of the manuscript. MB wrote the first draft of the manuscript. MB. BdJ, AS, and ME revised the manuscript.

### Conflict of Interest Statement

The authors declare that the research was conducted in the absence of any commercial or financial relationships that could be construed as a potential conflict of interest.
